# The dual role of p63 in cancer

**DOI:** 10.3389/fonc.2023.1116061

**Published:** 2023-04-27

**Authors:** Yongfeng Xu, Xiaojuan Yang, Qunli Xiong, Junhong Han, Qing Zhu

**Affiliations:** ^1^ Abdominal Oncology Ward, Cancer Center, West China Hospital of Sichuan University, Chengdu, Sichuan, China; ^2^ State Key Laboratory of Biotherapy and Cancer Center, Frontiers Science Center for Disease-Related Molecular Network, West China Hospital, Sichuan University, Chengdu, China

**Keywords:** p63, p53, DNA damage, cancer progression, protein-protein interactions (PPI), stem cell

## Abstract

The p53 family is made up of three transcription factors: p53, p63, and p73. These proteins are well-known regulators of cell function and play a crucial role in controlling various processes related to cancer progression, including cell division, proliferation, genomic stability, cell cycle arrest, senescence, and apoptosis. In response to extra- or intracellular stress or oncogenic stimulation, all members of the p53 family are mutated in structure or altered in expression levels to affect the signaling network, coordinating many other pivotal cellular processes. P63 exists as two main isoforms (TAp63 and ΔNp63) that have been contrastingly discovered; the TA and ΔN isoforms exhibit distinguished properties by promoting or inhibiting cancer progression. As such, p63 isoforms comprise a fully mysterious and challenging regulatory pathway. Recent studies have revealed the intricate role of p63 in regulating the DNA damage response (DDR) and its impact on diverse cellular processes. In this review, we will highlight the significance of how p63 isoforms respond to DNA damage and cancer stem cells, as well as the dual role of TAp63 and ΔNp63 in cancer.

## 1 Introduction

In most human malignancies, tumors develop through a series of genetic alterations, in contrast to the normal function of the p53 family gene (1–3); this procedure was identified as a tumor-driven progression to promote invasion, proliferation, cell survival, and drug resistance (4–6). Moreover, owing to the similar structure of p53 family members, p63 shares the function of p53 (e.g., activation of the apoptosis-related signal pathway in response to genome stress) (7–10), and the p53 homolog p63 is also capable of binding to the majority of p53-responsive promoters and initiating transcription (e.g., p21, Bax, MDM2, etc.) in development and homeostasis (11–16). There is increasing evidence that p53 and p63 can modulate resistance to cancer chemotherapy and DNA damage (17–19). Interestingly, some reports revealed that p63 is expressed in two main multiple isoforms, which often play very opposite functions in cancer progression.

TAp63 (a subtype of p63), widely known as a synergistic effector with p53, promotes cancer cell apoptosis after chemotherapy and is involved in cell cycle arrest, apoptosis, and DNA repair (20–23). In contrast, oddly, ΔNp63 (another main subtype of p63) serves more like an oncogene, presenting a phenotype to resist chemotherapy, inducing cell proliferation, and driving stem cell formation (24, 25). In addition, some reports also indicated that TAp63^-/-^ mice have an increasing number of breast hyperplastic cells with highly disordered, polarity defects, resulting in fragile skin, blisters, wounds that never heal, and alopecia. Nevertheless, ΔNp63^-/-^ transgenic mice showed a significantly accelerated keratinocyte differentiation through direct regulation of the Notch signaling pathway. This intricate phenotype relies on the defective proliferation and senility of dermal and epidermal precursors and indicates that both TAp63 and ΔNp63 may play roles in the development of skin stem cells. Why different subtypes of the same molecule play very different molecular and functional different molecular and functional. Hence, in this review, we focus on the latest developments in comprehending the regulatory network through which two p63 subtypes could potentially modulate molecular signaling pathways.

## 2 Structural features and biological functions of the p63 protein

**Figure 1:**
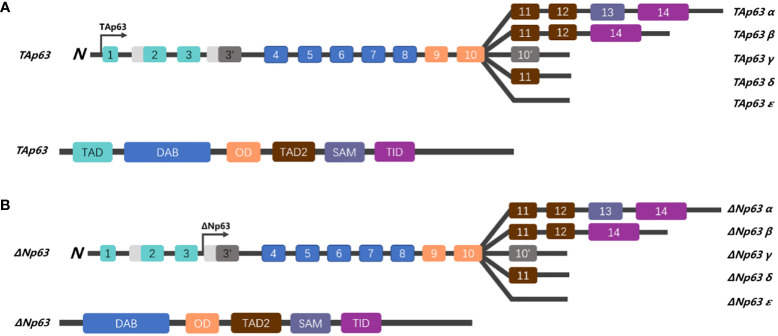
Schematic representation of the gene structure of p63. **(A)** The human p63 gene is located on chromosome 3q27 and spans over 250 kb, comprising 14 exons. Alternative splicing generates five isoforms (α, β, γ, δ, and ε), which differ in their C-terminus. The TAp63 isoforms, which contain a trans-activating domain (TAD), are encoded by exons 1, 2, and 3. **(B)** While ΔNp63 isoforms lack the TAD, TAp63 and ΔNp63 share a DNA binding domain (DBD), oligomerization domain (OD), sterile alpha domain (SAM), and a transactivation inhibitory domain (TID) in the C-terminal region.

The p53 family members have a series of similar gene frameworks and are constituted by three main domains: an N-terminal transactivation domain (TAD), a central DNA-binding domain (DBD), and an oligomerization domain (OD) (26, 27). Those highly similar homeodomains among p53 family members allow binding transactivation of the same gene promoters. Similar to the structure of p53, p63 is capable of recognizing and binding to the TAD of p53 response elements two or more tandem repeats of RRRCWWGYYY and initiating transcription of various genes (elaborated below), hence replacing a portion of the functions with p53 (such as cell cycle arrest and activation of apoptosis) (28). Unlike p53, p63 has two different promoters: the first promoter drives the transcription of TAp63, while the second promoter triggers the transcriptional activation of ΔNp63 isotypes; therefore, the p63 protein can be divided into two isoforms, depending on the different domains. The TA forms include the TAD, whereas the ΔN isoforms do not (**Figure 1**) (27).

It is generally understood that the two subtypes of p63 have different functions. Some researchers believe that the TAp63 subtype has a longer acidic N-terminal trans-transcriptional activation region similar to p53, which can undoubtedly transactivate p53-related downstream target genes, arrest the cell cycle, and induce apoptosis, resulting in p53-like biological effects and function as a tumor suppressor (29–31). In contrast, the biological characteristics of ΔNp63 are opposite to those of the TAp63 isomer. ΔNp63 lacks the TAD and, therefore, cannot induce transcription, loses the transactivating function of p53 downstream target genes, and has opposite results to p53-related cell cycle arrest and apoptosis (32–34). Furthermore, both TAp63 and ΔNp63 can compete for the DBD to directly activate or inactivate downstream cell proliferation or apoptosis (such as the p53–p21 signaling pathway, which can inhibit Cyclin E/Cdk2 to mediate cell senescence and restrain cell proliferation) (26, 35–37). In summary, similar to p53, TAp63 plays a role in cancer suppression; in contrast, ΔNp63 plays opposite roles in cell cycle regulation and apoptosis.

## 3 P53 prion-like behavior affects p63

The tumor protein p53 is a main transcriptional regulator in multiple significant signaling and programmed cell death pathways, in response to diverse genome stresses, such as DNA mutation, reactive oxygen species (ROS) injury, oncogene activation, or others, affecting a series of cellular processes, including DNA repair, cell cycle arrest, senescence, apoptosis, and differentiation (38–40). However, once the p53 pathway has been hijacked, it will not fulfill the normal obligations of the “guardian of the genome” to induce aging, or apoptosis in response to genotoxic stress. What was worse, mutated p53 does not perform a normal protective function but promotes cell proliferation and anti-apoptosis, causes tumor formation, and even leads to chemotherapy resistance and radiotherapy resistance for cancer survival (6, 41–43). Specifically, according to data from The Cancer Genome Atlas (TCGA) platform, more than half of cancer patients experienced mutation of p53 (44). Most p53 mutations are located within the DBD (96–293 aa) at several hotspots, such as R175, R248, R273, and R282, leading further to “gain of function” (GOF), which plays a significant role in promoting cancer progression and chemoresistance (45–48).

In contrast to the comparatively infrequent mutations of the p63 gene, p53 is often downregulated or mutated in tumors (49). To be specific, more than 95% of p53 mutations lie in the DBD, a mutated hotspot located in the DBD (such as R175H), resulting in structural instability and polarity disorder (50). In fact, when the DBD is mutated, hydrophobic core fragments 251–257 are exposed, resulting in p53 aggregation. Even worse, aggregation of mutant p53 (mut-p53) not only interfered with the transcriptional activity of wild-type p53 (wt-p53) in the nucleus but also congregated to p63 and p73, leading to coprecipitation, misfolding, and loss of normal function of wt-p53, p63, and p73, contributing to tumorigenesis. Interestingly, wt-p53 did not interact detectably with either p63 or p73, but mut-p53 coimmunoprecipitated with wt-p53, p63, and p73, then mut-p53 significantly counteracted the wt-p53/p63-induced growth inhibition (51–55).

Intriguingly, these aggressive behaviors of mut-p53 render us reminiscent of prion-like properties. Similar to prion, the infectious nature of mut-p53 is characterized by (i) mutated p53 attacking the wt-p53 protein, (ii) misfolding and assembly into amyloid granules, (iii) nucleic acid free, and (iv) propagating to other cells like virus (56).

Because of this prion-like behavior, mut-p53 amyloid can possess a “seeding” capacity and transmit to other cells. Once mut-p53 is internalized in other cells, the amount of mut-p53 amyloid seeds can misfold and aggregate with wt-p53/p63/p73 (56), acting similarly to amyloid-associated diseases, such as Alzheimer’s and Parkinson’s disease (57, 58). Meanwhile, numerous studies have established that the aggregation of mut-p53 assembled TAp63/TAp73, and aggregated and inactivated them into perinuclear amyloid oligomers, and this aggregation behavior can be suppressed by treatment with nocodazole, a small chemical that disrupts microtubule assembly (51, 54, 59, 60). This prion-like behavior of oncogenic mut-p53 provides an explanation for its binding and inactivation of TAp63, which is involved in the regulation of progression and apoptosis, and increases the drug resistance and invasion ability of tumors.

The identification of the p21 gene as a target of induction by wt-p53 protein was the first one on record (61). Cyclin E and cyclin A/CDK genes are associated with the p53-dependent cell cycle arrest that occurs at the G1/S transition in response to various factors, such as oncogenes or chemotherapies. However, once wt-p53 is affected by some factors and mutates, mutant p53 is highly susceptible to misfolding, leading to its accumulation as large aggregates inside the cell, which results in the loss of its physiological function as a tumor-suppressor protein.

**Figure 2:**
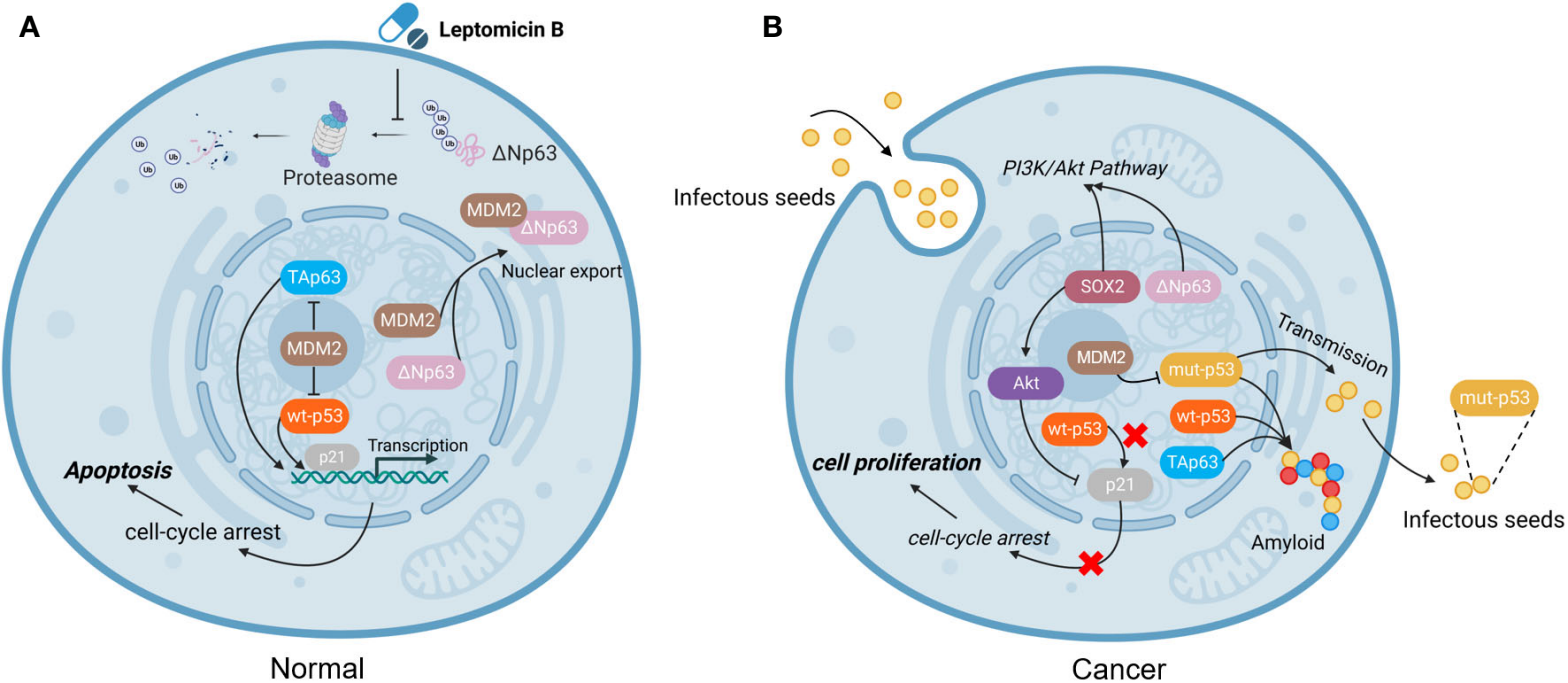
The function of mutated p53. **(A)** In normal cells, MDM2 plays an important role in regulating apoptosis. MDM2 binds to ΔNp63 and promotes its entry into the cytoplasm for degradation *via* proteasome, which can be blocked by the drug Leptomycin B (63). **(B)** In tumor cells, mut-p53 behaves in a prion-like behavior, not only possessing “seeding” ability and spreading to other cells, but also forming amyloid aggregates with TAp63 and wt-p53, leading to inactivation and promoting cell proliferation. However, MDM2 can block the aggregation of mut-p53, prevent mut-p53 from binding to TAp63, and alleviate mut-p53-related suppression to TAp63 (13, 64). (Created with Biorender.com).

The amyloid structures of p53 can penetrate cells and trigger the formation of amyloid aggregates of endogenous wt-p53 and TAp63. Loss of the native function of genes can cause genomic instability, which is a critical contributor to cancer development. What is worse, the results confirmed that p53 amyloid can be internalized and has the ability to “seed” the formation of amyloid structures. Once inside the cell, even a small number of mut-p53 amyloid seeds can promote the formation of amyloid aggregates of wt-p53 and TAp63, in a manner akin to prions. This templating ability of p53 fibrillar seeds renders wt-p53 and TAp63 nonfunctional and keeps ΔNp63 relatively highly expressed (62). Hence, we hypothesized that mut-p53 amyloids might spread between cells in a prion-like manner, which could have detrimental effects on cellular integrity. This spread could lead to the pervasive loss of wt-p53 function in tissues, effectively converting the guardian of the genome, p53, into a prion-like protein (**Figure 2**).

As we mentioned above, mut-p53 aggregated with wt-p53 and TAp63, resulting in the loss of their surveillant function in cancer formation. In addition, mut-p53 bound more efficiently to TAp63 than to the corresponding ΔNp63 isoforms (52), TAp63 and ΔNp63 are a well-known pair of contradictions, and TAp63 acts as a transcription factor, both functionally and structurally similar to the tumor suppressor p53, to induce cell apoptosis and suppress tumorigenesis, and is involved in the regulation of cell cycle arrest for DNA damage and aging. On the contrary, ΔNp63 acts in the opposite manner with wt-p53 and TAp63, and overexpression of ΔNp63 induces the accelerated growth of transformed cells *in vitro* and *in vivo*. Immunoprecipitation experiment showed that ΔNp63 interacts effectively with wt-p53 but not mut-p53, then ΔNp63 continuously inhibits p53-mediated transactivation by competitive binding of transcription factors to the same promoter regions of the target sequence (65). Interestingly, in combination with the above-reported prion-like behavior of mut-p53, it binds with TAp63 but not ΔNp63, resulting in amyloid precipitation, which makes TAp63 unable to perform its normal tumor suppressor function and renders a relatively high expression level of ΔNp63 in mut-p53 cells.

## 4 Dual role of p63

### 4.1 P63 and DNA damage and aging

#### 4.1.1 TAp63 in DNA damage and aging

The occurrence of senescence is a multi-procedural process; cells suffered from DNA damage under the genome pressure (such as ionizing radiation, ROS, and chemical agents). Unchecked DNA damage is an unexpected event for cells, resulting in mutation, chromosomal breakage, and cell cycle halting during S-phase; however, loss of the ability to monitor cell cycle checkpoints induced by DNA damage is a hallmark of cancer cells (66).

TAp63 as a widely known carcinogenesis and progression suppressor, nevertheless inactivate TAp63 by forming amyloid particles with mut-p53 or other signs of progression, including chemoresistance and antagonistic with senescence (67). Furthermore, some researchers believe that forced expression of TAp63 results in a synergistic effect to induce chemotherapeutic treatment-relative apoptosis in hepatoma cells. Additionally, Gressner’s group found that TAp63 has the ability to activate both death receptor- and mitochondria-mediated apoptosis pathways, and both signaling processes are renowned for reinforcing sensitivity to chemotherapy; on the contrary, blocking TAp63 function leads to enhanced chemoresistance (68).

Meanwhile, p63 has also been described to play a significant role in regulating the apoptotic response following DNA damage agents (69–72). Some reports indicated that ΔNp63 expression levels decreased after treatment for 24 h with ultraviolet radiation (73); in contrast, exposure to cisplatin for 24 h induced DNA damage, and although the total TAp63 protein level did not change, the phosphorylation level of TAp63 increased. This genomic injury activated phospho-TAp63^Ser395^, induced the SAPK/JNK signaling pathway, and triggered apoptosis in oocytes and granulosa cells. In addition, Gressner’s group also measured the expression levels of the CD95, TNF-R, and TRAIL-R cell death-related NF-κB pathways, and found that stimulation of TAp63 can trigger each of these death receptors and consequently sensitize tumor cells toward apoptosis (68).

TAp63 proteins are degraded *via* the ubiquitin–proteasome pathway under normal cellular circumstances (74, 75). While other reports also indicated that genotoxic agents, including ultraviolet (UV) irradiation, actinomycin D, bleomycin, and etoposide, led to elevated expression of TAp63 protein levels, the interaction with Cables1 is responsible for the stabilization of the TAp63 isoform structure, which enables apoptosis of cells in response to genotoxic agents (76). According to this, it appears that under the stress of DNA damage, cells promote high expression of Tap63 and stabilize the structure of Tap63, thereby promoting a series of reactions that induce cell apoptosis.

#### 4.1.2 ΔNp63 in DNA damage and aging

The primary p63 isoform, known as “ΔNp63,” has been shown to impede the transactivation of p53, TAp63, and TAp73 by the specific formation of inhibitory heterogenous complexes to competitively bind the promoters and affect their downstream target genes (26, 27, 36, 77). ΔNp63 is often found highly expressed in various cancers; ΔNp63 protein levels are significantly repressed after UV irradiation, and exotic expression of ΔNp63 alleviates the UV-induced apoptosis (65, 78). In addition, ΔNp63 also triggers a variety of survival signaling cascades, for example, the epithelial growth factor receptor (EGFR), transforming growth factor (TGFβ), and hepatocyte growth factor receptor (HGFR) pathways to drive tumor invasiveness and metastasis (79–82), and activate a set of DNA damage repair-related genes [such as CDK12 (83) and SMG1 (84) proteins], thus promoting cancer cell survival and proliferation. Meaningfully, the kinases CDK12 and SMG1 are recruited to chromatin upon ultraviolet (UV) irradiation only in the presence of ΔNp63 (85), suggesting ΔNp63 as a trigger in the DNA repair signaling pathway. Moreover, reduced ΔNp63 level promoted recruitment of the DNA damage responsive proteins (e.g., FANCI and Lsh) to chromatin, and promoted the expression of γH2A.X, indicating ongoing DNA damage and repair (85). Additionally, ΔNp63 is consequently phosphorylated after DNA damage by ATM, CDK2, and p70s6K. Exposure to DNA damage (such as cisplatin)-induced phosphorylation of ΔNp63 (S385, T397, and S466) leads to a rapid degradation of ΔNp63 protein levels in cancer cells, and results in the transfer of cisplatin-resistant cells into cisplatin-sensitive cells (75). ATM, CDK2, and p70s6K are key regulators in the cellular response to DNA injury, whose activation/homodimerization causes ATM to bind to and phosphorylate its sequencing protein targets and impact DNA repair, apoptosis, and cell cycle checkpoints (86, 87). Of particular importance, although ΔNp63 was degraded under the exposure of genotoxic substances, interaction with YAP1 to stabilize ΔNp63 protects cancer cells from UV-induced apoptosis (88). Liefer et al. also found that the number of apoptotic cells in ΔNp63 transgenic mice decreased by 40%–45% compared with non-transgenic mice under the exposure of UV (69). Hence, ΔNp63 inhibits receptor-mediated and chemotherapy-induced mitochondrial apoptosis pathways, and may assist in predicting cancer cells in response to various genotoxic stresses (89).

**Figure 3:**
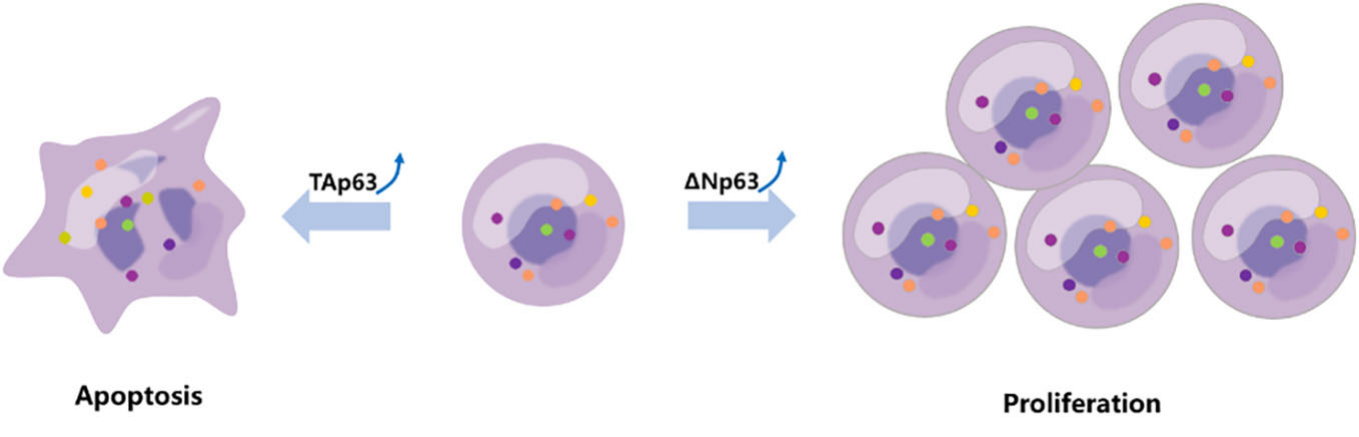
TAp63 and ΔNp63 exert different roles in response to chemotherapy. The TAp63 isoform is frequently associated with tumor suppression involved in cell cycle arrest, apoptosis, and DNA repair. In contrast, the ΔNp63 isoform serves as an oncogene, repressing proapoptotic genes, increasing chemotherapeutic resistance, and inducing cell proliferation.

Previous interesting studies presented that cancer cells exposed to genotoxic stress agents (such as UV-irradiation and etoposide) accumulated expression of TAp63 (90). Furthermore, TAp63 is degraded *via* the lysosomal degradation pathway under normal cellular circumstances but stabilized under genotoxic stress (91). Another report showed that under the exposure to UV-B irradiation, increasing genotoxic pressure mediated the downregulation of ΔNp63 protein levels (69). Together, these two different P63 family proteins regulate different cell homeostasis under genotoxic stress (**Figure 3**).

### 4.2 P63 and chemotherapy resistance

#### 4.2.1 TAp63 in chemotherapy resistance

Tumor recurrence after chemotherapy is still a troublesome problem for physicians. This is frequently brought out by the multidrug resistance (MDR) reaction to chemotherapy drugs (92). The effectiveness of malignancies in response to chemotherapeutic agents depends on many factors, most of which are currently unknown. Some scholars believe that the molecular mechanism of resistant response occurs through DNA damage repair and subsequent p53 overexpression, further induction of apoptosis, or cell cycle arrest (93).

P53, as a guidance of the genome, protects cells from radiation or other genotoxic stresses and ultimately causes cell apoptosis. However, the mutant form of p53 can confer resistance to chemotherapy-induced apoptosis, thereby reducing tumor cell susceptibility to cell death. As we mentioned above, the prion-like behavior of mut-p53 binds with TAp63 but not ΔNp63, resulting in amyloid precipitation, which makes TAp63 unable to perform its normal tumor suppressor function and induce the apoptosis of tumor cells under chemotherapy. Interestingly, Flores et al. showed that, compared with p53^+/-^ mice alone, p53^+/-^ and TAp63^+/-^ transgenic mice spontaneously formed cancer at a dramatically higher incidence. Moreover, p53^+/-^ and TAp63^+/-^ double knockdown mice presented a shorter life span, formed multiple primary tumors (such as bladder, breast, and esophageal cancers), and a much higher tendency to promote metastasis of cancer, and were linked to organismal aging (94). Moreover, another independent research reported that like p53, the TAp63 isoform is an outstanding mediator to monitor tumorigenesis and aging *in vivo*; p53^-/-^ and p63^-/-^ compared with p53^-/-^ and p73^-/-^ mice presented a higher resistance to DNA damage-induced apoptosis, but transfection of TAp63 into p63^-/-^ mutant mice caused a significant increase in doxorubicin-induced cellular senescence (70). In addition, Guo et al. also found that high expression of TAp63 is an excellent indicator of senescence, independent of p53, prohibits Ras-mediated cancer development, and promotes doxycycline-induced cellular senescence, whereas loss of TAp63 results in aggressive tumor phenotypes, accelerated proliferation, and relieved senescence in cancer cells (37). Furthermore, TAp63 is degraded *via* the lysosomal degradation pathway under normal cellular circumstances but stabilized under genotoxic stress (91).

Taken as a whole, the TAp63 isoform may limit tumor growth by controlling senescence through p53-independent mechanisms. Inhabitation of senescence is one of the hallmarks of cancer, therefore reactivating senescence in tumor cells, especially those resistant to genotoxicity-induced death, so TAp63 should be considered as a significant mediator in senescence that inhibits tumorigenesis and provides a new foundation on anticancer treatment.

Previous interesting studies presented that cancer cells exposed under genotoxic stress agents (such as UV irradiation and etoposide) accumulated expression of TAp63 but mediated downregulation of ΔNp63 proteins (69, 90). Thus, another subtype of p63, ΔNp63, should also be discussed in the next section.

#### 4.2.2 ΔNp63 in chemotherapy resistance

The p53 family member p63 plays an important role in the cell cycle checkpoint. The ΔN isoform of p63 (ΔNp63), a dominant inactivated form of P63, promotes the proliferation of tumor cells by inhibiting the transcription of the cell cycle regulators (p21, cyclin B2, and cdc2) that influence downstream signaling pathways and resist apoptosis, and can be considered as a prognostic indicator (36, 95–97).

**Figure 4:**
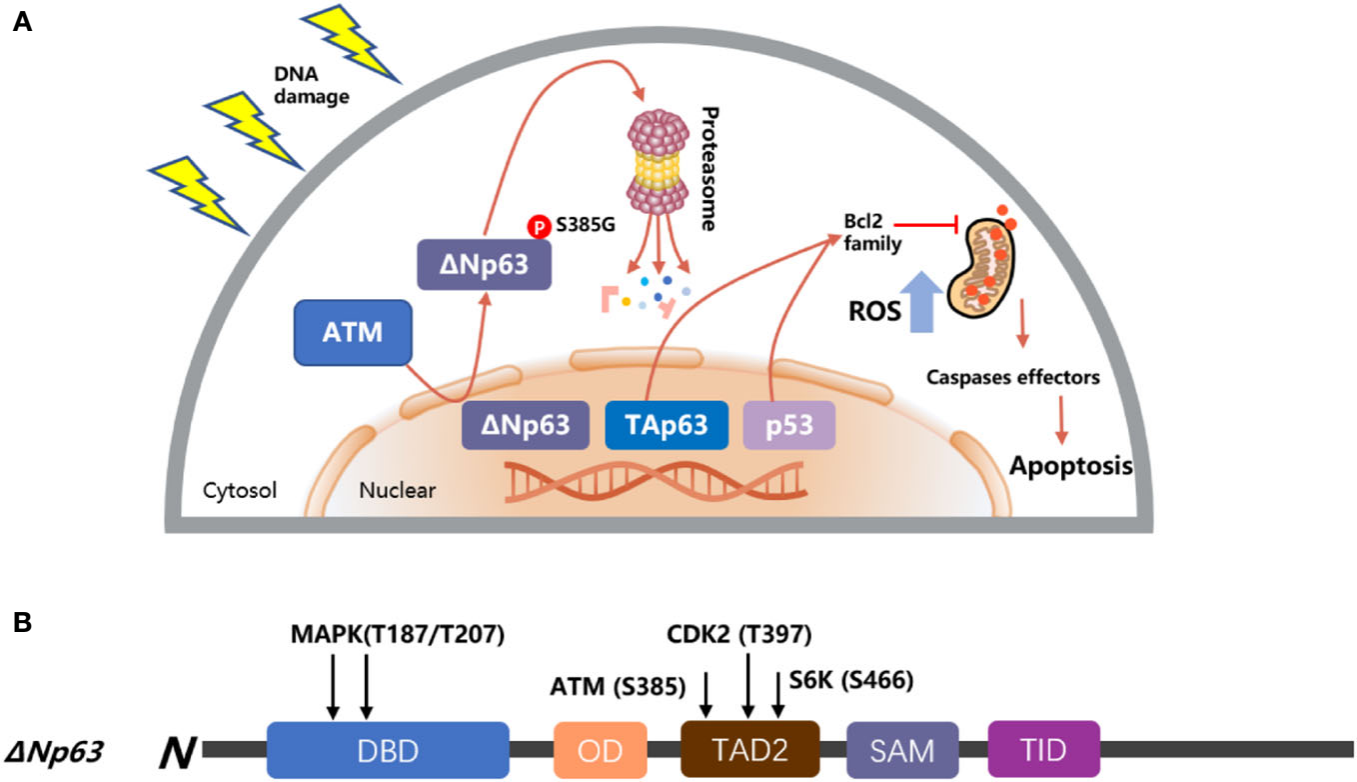
Model of p53 and p63 in response to DNA damage. **(A)** TAp63 and p53 induce apoptosis pathways to activate the mitochondrial cascades, and ΔNp63 is phosphorylated at S385G (p-ΔNp63^S385G^) by ATM and degraded following DNA damage. **(B)** The modular structure of ΔNp63 with putative phosphorylation sites. The arrows indicate the newly identified phosphorylation sites for MAPK (T187/T207), ATM (S385), CDK2 (T397), and p70s6K (S466) kinases (75, 105, 106).

A number of reports have shown that the expression of ΔNp63 is associated with cancer proliferation and drug resistance (98–100). The ΔNp63 protein level has a negative correlation with the concentration of bortezomib, and silencing ΔNp63 significantly reduces the volume of cancer and enhances the survival of mice treated with bortezomib. In contrast, mice treated with bortezomib showed a higher cancer load and a shorter life span following forced ΔNp63 expression (101). Another report indicated that knockdown of ΔNp63 in BRAFi-resistant cells promoted resensitization of these resistant cells in response to vemurafenib and directly enhanced the activation of p53-dependent mitochondrial apoptotic pathways; on the contrary, overexpression of ΔNp63 promoted cell proliferation and enhanced cell resistance to genotoxicity-induced apoptosis (102, 103), and resistance to MAPK inhibitors (18). In squamous cell carcinoma of the head and neck (HNSCC), after the treatment of cisplatin, ΔNp63 is phosphorylated at S385G (p-ΔNp63^S385G^) by ATM and degraded following DNA damage (104); then, ΔNp63 also downregulates the expression of mir-181a, mir-519a, and mir-374a, leading to a series of mRNAs involved in apoptosis, rendering cancer cells more sensitive to DNA damage agents (**Figure 4**) (75, 107, 108). Moreover, ΔNp63 also activated phospho-EGFR (Y1086); promoted EGF-mediated activation of ERK, Akt, and JNK signaling; stimulated cancer proliferation, motility, and invasion; and enhanced resistance to cisplatin-induced apoptosis; on the other hand, when a missense mutation was introduced into the ΔNp63 DBD at position 202, it will downregulate the expression of EGFR (109, 110). In order to promote the accumulation of ΔNp63 in cancer, the deubiquitylate USP28 stabilizes ΔNp63 by counteracting its proteasome-mediated degradation, promoting cancer cell survival under the treatment of chemotherapy (99). Furthermore, some findings highlight that p63 plays an important role in controlling ROS. Overexpression of ΔNp63 cooperates with the BCL-2 family to prevent etoposide-induced ROS accumulation, leading to ferroptosis independent of p53 (111, 112). Moreover, a report also indicated that with the increased dose of H_2_O_2_-induced ROS, ΔNp63 increased gradually (113), and this procedure provides a way for tumor cells to inhibit oxidative stress-induced cell death and promote survival.

Therefore, it can be concluded that the TA-type ΔN homolog of the P63 gene has the opposite biological function in chemotherapy response. The TA-type homolog has the potential activity of a tumor suppressor gene while the ΔN-type homolog has the function of an oncogene to promote the survival of cancer cells.

### 4.3 Stem cell

#### 4.3.1 TAp63 in stem cell

It has been hypothesized that only a small group of cancer cells, known as cancer stem cells (CSCs) or cancer-initiating cells (CICs), capable of infinite self-renewal capacity, rapid reproduction, and resistance to chemotherapy, are responsible for tumor initiation, progression, and metastasis (114–116). Furthermore, p63 has been implicated in body development, it is highly expressed in the proliferating basal cell layer, which contains a large number of epithelial progenitor cells responsible for normal replacement function of cells in tissues such as skin, prostate, eyelid, and jaw. Therefore, p63 may be involved in the maintenance of stemness and serve as a potential marker for identifying stem cells (117–121).

Since TAp63 shares the abilities of the “guardian of the genome” p53 to induce cell cycle arrest and apoptosis, TAp63 may thus act as a tumor suppressor. Meaningfully, the knockdown of TAp63 can significantly affect the function of the cells and even GOF to promote cell proliferation. Some reports have indicated that TAp63 participated in the regulation of stem cells *via* transcriptional regulation of LKB1, further affecting the Hippo pathway effector TAZ, which has previously been demonstrated to have crucial functions in the progression of stem cells and metastasis. Loss of regulation of LKB1 in TAp63-deficient mammary epithelial cells resulted in a loss of Scrib expression and activation of the Hippo pathway through TAZ and a subsequent loss of cell polarity and accumulation of cancer stem cells (122, 123). In addition, some animal studies have confirmed the role of TAp63 in tumor stemness suppressor. TAp63^-/-^ mice showed significantly increased proliferation and self-renewal, resulting in overproliferation of stem cells; however, as normal stem cells are not immortalized in proliferation progress, this procedure will exhaust normal adult stem-cell function and result in depletion of normal stem cells, and eventually resulting in TAp63^-/-^ mice having fragile skin, blisters, wounds that never heal, and alopecia (124–126). Furthermore, another report indicated that TAp63^-/-^ mice have an increasing number of breast hyperplastic cells with highly disordered and polarity defects, and the TAp63^-/-^ mice had 75% ± 1% Ki67-positive cells in the mammary gland, while WT mice only had 38% ± 2% Ki67-positive cells in the mammary gland. Moreover, 8% of TAp63^-/-^ mice also spontaneously form mammary adenocarcinoma at 9–16 months of age, and TAp63^-/-^ mice have also been shown to express high levels of Sox2 and BMP4, which are known markers for cancer stem cells (67, 123).

#### 4.3.2 ΔNp63 in stem cell

Very interestingly, unlike the downregulation of TAp63 to promote stem cell proportion, ΔNp63 acts like an oncotarget promoter to enhance cell proliferation, directly interacts with the Hippo effector YAP1, and is a mediator of YAP1 function to promote cancer cell spheroid formation, invasion, migration, and enhance cancer stem cell survival (127, 128). Moreover, ΔNp63 acts as an oncogene that positively participates in the Hedgehog signaling pathway by directly binding to Shh, Gli2, and Ptch1 gene regulatory regions and influencing stemness, contributing to enhancing CSCs’ self-renewal potential (129). In addition, ΔNp63 increases the expression of the Wnt receptor Frizzled 7, thereby combining with β-catenin to enhance Wnt signaling, which leads to promotion of normal mammary stem cell activity and tumor-initiating activity in the basal-like subtype of breast cancer (130, 131). Several additional *in vitro* and *in vivo* experiments also suggest that ΔNp63 drives the stem cell formation and differentiation in normal tissues. SETDB2 interacts with ΔNp63 and methylates and stabilizes the ΔNp63 protein, and SOX2 activates ΔNp63 by directly binding the enhancer site and rescued the cancer stem cell maintenance (132, 133). ΔNp63^-/-^ transgenic mice showed a significantly accelerated keratinocyte differentiation through direct regulation of the Notch signaling pathway (134). Liu et al. identified that ΔNp63 was upregulated in 100 of 173 (58%) breast cancer patients and was associated with poorer survival in patients with ER-/HER2+ breast cancer (135).

Moreover, many researchers currently recognize that CSCs are characterized by high expression of CD29, CD44, CD82, or CD133, which are associated with tumor progression and stemness in various cancers (136–140). In prostate cancer, ΔNp63, as a key regulator of CSC-related genes, cooperates with CD82 and is involved in tumor metastatic adhesion (141, 142). Additionally, overexpression of ΔNp63 promotes the expression of CD44 through an indirect way in HNSCC (143). In another report, according to the expression level of CD29, Li et al. divided the breast cancer cell population into CD29 high- and low-expression groups (CD29^high^ and CD29^low^) and found that TAp63 was highly expressed in CD29^low^ cells; in contrast, ΔNp63 was highly expressed in CD29^high^ cells (144). Meng’s group also identified that ΔNp63 directly activates Notch signaling pathway to induce cancer cells to acquire CSC-like properties, and the expression levels of ΔNp63 were positively correlated with CD133 to affect the self-renewal capacity of cancer cells (145). Hence, ΔNp63 could be considered a biomarker of certain epithelial stem cells and CSCs, and understanding the relationship between p63 isoforms and CSCs is helpful to understand the occurrence and development of tumor cells.

### 4.4 Posttranslational modifications of p63

Considering the great influence of post-transcriptional modification on protein function and structure, p63-related interactome is a significant parameter of the p73 activity (**Table 1**). P63 can be posttranslationally regulated by RNA-binding proteins (RBPs) RBM24, RPM38, and HuR *via* mRNA stability and protein translation (163–165); to be specific, RBM24 has the ability to bind to multiple sites within the 3’ untranslated region of p63 and destabilize the transcript, resulting in decreased p63 expression levels. Ectopic expression of RBM24 shortens the half-life of both TAp63 and ΔNp63 mRNA levels. This is due to RBM24 binding to multiple regions in the 3′UTR of the p63 transcript, which is essential for TAp63 expression. The RNA-binding domain in RBM24 is composed of two RNA recognition submotifs, RNP1 and RNP2, and in the absence of either RNP, RBM24 cannot bind to p63; thus, the RNA-binding domain of RBM24 is essential for binding to the p63 transcript, which leads to the inhibition of p63 expression. Other types of kinases have also been found to be involved in the activation process of Tap63, such as Cables1, TLR3, PML, and PlK1. The activation process of ΔNp63 also involves the participation of many other kinases, including ATM (75), CDK2 (75), HIPK2 (166), p38 (167), p70s6K (75), and Raf1 (168).

**Table 1:** Interacting partners of p63 isoforms and their effect on p63 function.

Protein interactor	P63 isoforms	Outcome	References
Wild-type p53	ΔNp63	Binding of wild-type p53 to ΔNp63 isoforms results in the degradation of p63.	(65)
Mutant p53	TAp63	Inhibition of TAp63-induced apoptosis.	(13, 146)
P21	ΔNp63 and TAp63	Cell cycle control and the proliferative potential of epidermal progenitor cells.	(14)
MDM2	ΔNp63 and TAp63	MDM2 competes with TAp63 for binding to mutant p53 and relieves the inhibition of TAp63 activity by mutant p53. The conserved FWL motif in the TA domain of TAp63 serves as a binding site for MDM2, promoting the degradation of TAp63. In addition, MDM2 can bind to ΔNp63 and promote its degradation through the proteasome pathway in the cytoplasm.	(13, 64)
BAX	TAp63	TAp63 induce apoptotic signaling proteins and require BAX expression and function for its effects.	(11)
YAP1	ΔNp63	Interaction with YAP1 to stabilize ΔNp63 and protect cancer cells from ultraviolet-induced apoptosis.	(88)
Cables1	TAp63	Stabilization of the TAp63 isoform structure under the exposure of genotoxic agents.	(76)
SETDB2	ΔNp63	Methylated and stabilized ΔNp63 protein.	(133)
AIP4	ΔNp63 and TAp63	Both proteasomal and lysosomal inhibitors inhibit p63 degradation upon Itch/AIP4 overexpression.	(147)
SOX2	ΔNp63	Both help maintain the immature precursor of squamous epithelia and are involved in the process of carcinogenesis.	(148)
miR-574, miR-720, and miR-203	ΔNp63	ΔNp63 maintains the proliferative ability of the cell by repressing the expression of miR-574, miR-720, and mir-34a.	(149)
SP-A	Unknown	P63 may play a role in the movement of SP-A from the endoplasmic reticulum to the plasma membrane.	(150)
TAp73	ΔNp63	Blocking TAp73 ability to transactivate bcl-2 family members and to induce cell apoptosis.	(151, 152)
IRF6	ΔNp63	Periderm development and palatal fusion.	(153)
NRF2	ΔNp63	Control of epidermal renewal.	(154)
Cables1	TAp63	Protect TAp63 from proteasomal degradation.	(76)
Cdc20	ΔNp63	Cdc20-induced degradation of ΔNp63.	(155)
KMT2D	ΔNp63	Maintenance of epithelial progenitor gene expression.	(156)
iASPP	ΔNp63	Regulation of skin development and epithelial homeostasis.	(157)
c-Rel	ΔNp63	Affecting NF-κB complexes to promote proliferation of keratinocytes.	(158)
HK2	ΔNp63	Regulation of cancer metabolic reprogramming.	(159)
c-Abl	TAp63	c-Abl phosphorylates TAp63 on tyrosine residues (Tyr149, Tyr171, and Tyr289) and stabilizes TAp63	(160, 161)
c-Abl	ΔNp63	c-Abl regulates ΔNp63 protein stability by phosphorylation on Y55F, Y137F, and Y308F, and promoting ΔNp63 to bind with YAP to accelerate cancer cell proliferation	(162)

Significantly, after cisplatin treatment, in response to DNA damage, c-Abl kinase detects the signal and phosphorylates TAp63 on specific tyrosine residues (Tyr149, Tyr171, and Tyr289) and stabilizes TAp63, consistent with c-Abl nuclear accumulation toward apoptotic genes (160, 161). Moreover, repression of this process by imatinib, a BCR-ABL inhibitor used to clinically treat chronic myelogenous leukemia, results in the abolition of TAp63 activation and protection of mouse oocytes from cisplatin chemotherapy (160).

Interestingly, in cancer, c-Abl also regulates ΔNp63 protein stability by phosphorylation on Y55F, Y137F, and Y308F, and promoting ΔNp63 to bind with YAP to accelerate cancer cell proliferation (162). Taken as a whole, c-Abl phosphorylates and stabilizes TAp63 to perform its apoptotic function in normal cells under the exposure of chemotherapy, but, after the treatment of cisplatin in cancer cells, c-Abl phosphorylates and stabilizes ΔNp63 to promote cancer cell survival.

Furthermore, Hsp70 (heat-shock protein 70) and CHIP (C-terminus of Hsc-70 interacting protein) have been reported as critical switches for TAp63 and ΔNp63 ubiquitination and degradation (169, 170), and both Hsp70 and CHIP are involved in the process of ubiquitin ligase activity.

CHIP, as a cochaperone ubiquitin ligase, is a highly conserved ubiquitin E3 ligase containing a U-box domain responsible for chaperone partners. CHIP has an N-terminal tetratricopeptide repeat (TPR) domain involved in protein–protein interactions (PPIs) with Hsp70 and Hsp90. Moreover, CHIP has also been proven to conserve the ubiquitin E3 ligase of p53 (171), c-Myc (172), PRMT5 (173), and EGFR (174). Moreover, Wu et al. also proved that the stability of TAp63 and ΔNp63 is regulated by CHIP/Hsp70-mediated ubiquitin–proteasome degradation (170).

Hsp70 acts as a crucial switch to control the CHIP-mediated ubiquitination and degradation of both TAp63 and ΔNp63 isoforms. Hsp70 depletion by siRNA enhanced the interaction of CHIP with ΔNp63 but reduced the interaction with TAp63, thus promoting ΔNp63 degradation, increasing the expression of TAp63, and downregulating the expression of ΔNp63 in cancer cells. Furthermore, the author also used a small-molecule inhibitor of Hsp70, called Ver-155008, and a similar result has been observed in which an increase in ΔNp63 ubiquitination and an accompanying decrease in ΔNp63 protein levels suggest that Hsp70 is involved in CHIP-mediated p63 degradation. Thus, c-Abl, Hsp70, and CHIP seem to play a dual role in tumor and normal cells, and this vague condition in response to chemotherapy requires further studies to elucidate the potential mechanisms underlying these effects.

## 5 Discussion

The p53 family proteins exert a crucial dual role in cancer development and chemotherapy. P63, as a significant regulatory factor similar to p53, is involved in tissue proliferation and differentiation, acts as a transcriptional regulator of tumorigenesis, and is highly expressed in the basal cells where a majority of human epithelial neoplasm develop. Here, we reviewed the influence of two major p63 subtypes (ΔNp63 and TAp63) in pathological conditions, such as cancer stem cells, DNA damage, and drug resistance. Consistently, the balance between TAp63 and ΔNp63 isoforms appears to be important in regulating cellular fates, the ability of tumor suppressors vs. oncogenes, sensitivity vs. drug resistance, and apoptosis vs. proliferation. p63 is widely expressed in cancer tissues and is essential for the survival of cancer cells under the exposure of DNA damage agents. However, there is currently no antitumor drug that targets p63 in DrugBank or other databases. We also reflect on whether p63 is a potential therapeutic target in light of the significant role that p63 plays in the progression of tumors. Taken together, TAp63 isoforms can potentially emulate wt-p53 functions in cancer cells by promoting apoptosis in response to DNA damage, while ΔNp63 isoforms imitate the ability of mut-p53 to initiate cell proliferation and resist DNA replication stress. In this regard, estimating the rankings of the specific p63 isoforms in various cancer patients is of high relevance as it may have a promising impact on patient prognosis and therapeutic outcomes.

## Author contributions

Contributions: (I) Conception and design: YX; (II) Administrative support: QZ and JH; (III) Provision of study materials: XY; (IV) Collection and assembly of data: QX; (V) Data analysis and interpretation: YX; (VI) Manuscript writing: All authors; (VII) Final approval of manuscript: All authors.
